# Where’s the problem? Considering Laing and Esterson’s account of schizophrenia, social models of disability, and extended mental disorder

**DOI:** 10.1007/s11017-017-9413-0

**Published:** 2017-07-05

**Authors:** Rachel Cooper

**Affiliations:** Politics, Philosophy & Religion, Lancaster University, LA1 4YG Lancaster, UK

**Keywords:** Social model of disability, R. D. Laing, Schizophrenia, Extended mind, Internal location

## Abstract

In this article, I compare and evaluate R. D. Laing and A. Esterson’s account of schizophrenia as developed in *Sanity, Madness and the Family* (1964), social models of disability, and accounts of extended mental disorder. These accounts claim that some putative disorders (schizophrenia, disability, certain mental disorders) should not be thought of as reflecting biological or psychological dysfunction within the afflicted individual, but instead as external problems (to be located in the family, or in the material and social environment). In this article, I consider the grounds on which such claims might be supported. I argue that problems should not be located within an individual putative patient in cases where there is some acceptable test environment in which there is no problem. A number of cases where such an argument can show that there is no internal disorder are discussed. I argue, however, that Laing and Esterson’s argument—that schizophrenia is not within diagnosed patients—does not work. The problem with their argument is that they fail to show that the diagnosed women in their study function adequately in any environment.

Disorders are standardly thought of as being located within affected individuals. In the case of a patient with cancer, for example, a scan or knife can open up the patient’s body and reveal the tumour hidden within. We accept, of course, that disorders are caught up in causal networks that extend beyond the confines of the patient’s skin. Many disorders are caused by factors external to the patient (pollution, poisons, and germs). Many disorders go on to cause problems for others, apart from the patient (others may catch the disease). Still, we think of the disorder itself as having its site within the patient’s body. We think that there is some part of the patient that is not working as it should.

In this article, I compare and evaluate the account of schizophrenia offered by R. D. Laing and A. Esterson in *Sanity, Madness and the Family* (1964) [1], social models of disability, and views of mental disorder that might be developed from an extended cognition perspective. These are all ‘externalising’ accounts, in that they each advance the idea that at least some conditions, standardly considered disorders, should not be thought of as being located within the patient. I want to consider whether any sense can be made of such claims. What reasons might someone give for locating a putative disorder externally as opposed to internally? Are any of these accounts right?

The question of where putative disorders are located matters for a number of reasons. It has implications for the viability of different explanatory projects. Person-centred explanatory projects only make sense if disorders are sited within patients; there is no point looking to brain scans for the basis of schizophrenia, if it is not located in brains. There are also implications for the style of ameliorative attempts that might be appropriate. If problems are caused by some dysfunctioning element within a patient’s body then treatment with surgery or drugs may make sense. If, on the other hand, the source of difficulties is external then it is plausible that the environment should be changed. This has moral and political, as well as practical, implications. Thinking of a problem as caused by a disorder that is rooted in a dysfunction within an individual can act to exceptionalise, individualise, and depoliticise difficulties [2]. Think, for example, of the problem that some children are inattentive and restless in classrooms. Via diagnosing some individual children with attention deficit hyperactivity disorder (ADHD), this problem comes to be thought of as stemming from problems in a few children’s brains. In contrast, if the problems are not thought to be within the brains of certain dysfunctional children, the fact that some children are inattentive and restless may serve as an implicit criticism of someone else. Possibly the classroom is poorly designed? Maybe the teachers are not skilled enough?

There are also conceptual implications. Some claim that it is part of the concept of disease or disorder that the condition must be located internally to the patient.^1^ Even on accounts where this claim is not made explicit, the popular idea that disorders are ‘dysfunctions’ in bodily or psychological subsystems implies an internal location for disorders [6–11]. The D.S.M., the influential classification of mental disorders published by the American Psychiatric Association, distinguishes conflict that is symptomatic of mental disorders from conflict that is social or political on the basis that symptoms of mental disorder result ‘from a dysfunction *in* the individual’ [12, p. 20] (emphasis added). If it is part of the concept of disorder that disorders must be located within patients, then if some putative disorder cannot be sited within a patient, this goes to show that the problem is not a disorder at all. Rather than being a medical problem, the problem must be of some other kind—for example, social, political or economic.

In what follows, I first outline the three ‘externalising’ accounts to be examined. I then abstract from them and seek to discern what reasons in general there may be for locating a problem within or without an individual. I then turn to questions of evaluation—is there actually reason to think that certain conditions, normally considered disorders, might not be located within individual patients? To give a preview of what is to come, I shall conclude that in at least some cases, the claims of the proponents of the social model of disability, and of extended mental disorder, are plausible. Some putative disabilities and mental disorders are best not thought of as indicating that there is anything wrong within the diagnosed individual. On the other hand, I shall argue that Laing and Esterson’s argument does not succeed; they fail to show that schizophrenia should not be located within individuals. Still, although their account ultimately fails, considering it in detail is worthwhile for two reasons. First, Laing and Esterson’s book is so influential that examining exactly why their argument fails is worthwhile (and doing this is a secondary objective of this article). Second, while their argument fails, an argument that can be developed from it will work, and can be used to show that some putative mental disorders (though not schizophrenia) are best not thought of as rooted within the dysfunctioning minds of individuals.

## Three ‘externalising’ accounts

### R. D. Laing and A. Esterson’s *Sanity, Madness and the Family* (1964)

R. D. Laing was a prolific and controversial thinker who advanced a number of distinct claims throughout his career. Here, I am concerned only with the account of schizophrenia outlined by Laing and Esterson in their jointly-authored book *Sanity, Madness and the Family* (1964) [1]. The arguments of this book will be considered on their own merits, in isolation from Laing’s other works.

*Sanity, Madness and the Family* (1964) reports on a series of interviews conducted with women who had been diagnosed with schizophrenia and their families. The interviews were conducted in the late 1950s, somewhere in Britain (the location is not disclosed in the book to protect the identity of the patients). The researchers conducted hours and hours of interviews with each woman and various combinations of her family members—there might, for example, be eight hours of interviews with the woman alone; ten with her and her mother; eight with her, her mother, and her father; four with her father and mother; and three with her, her mother, father, and grandparents. The interviewers were interested in the ways in which the different family members communicated.

In the family interviews, the researchers noticed many examples of odd and unhelpful communications. They saw examples of what they termed ‘mystification’. The daughter might be told things like, ‘It’s best to stay at home’ or ‘Of course you’ve always been special’, with it never being made exactly clear what the speaker was hinting at. There were also examples of ‘invalidation’, whereby claims made by the daughter were constantly undermined. She might report wanting or feeling something, only to be told that really she did not. For example, whenever the daughter reported remembering something, she might be told, ‘Of course your memory’s never been good’. The interviewers also noted examples of ‘double binds’, in the sense introduced by the anthropologist Gregory Bateson [13]. A double bind is imposed on someone when they are subjected to contradictory demands. The contradictory demands are generally made at different levels of communication, for example, there may be a clash between what is spoken and the message conveyed by body language. In a classic example, a mother asks her child for a kiss while simultaneously pushing her away. The power relations are such that the ‘victim’ of the double bind is unable to directly comment on it. They cannot simply say to the perpetrator ‘You’ve indicated that you want me to do two contradictory things, what do you really want?’ Rather, the victim is left in an impossible situation—whatever they do, they cannot win.

On the basis of the interviews, the main claim made in *Sanity, Madness and the Family* is that schizophrenia is not ‘in’ the diagnosed patients.^2^ This conclusion is claimed to be justified by the fact that when the speech and behaviour of the women is viewed within the context of their families, it makes (more) sense:

We set out to illustrate by eleven examples that, if we look at some experience and behaviour without reference to family interactions, they may appear comparatively socially senseless, but that if we look at the same experiences and behaviour in their original family context they are liable to make more sense. [1, p. 12]

In some of Laing and Esterson’s cases, this claim seems plausible. The chapter on ‘The Abbotts’ reports on interviews with ‘Maya’ and members of her family. Maya has been diagnosed as having paranoid schizophrenia. In psychiatric interviews, she is reported to have various delusions; she has odd ideas about mind-reading, she thinks that others can read her thoughts, that they are constantly discussing her, and so on. When the interviewers go to Maya’s home and speak with her and her parents, they notice that her mother and father seem to be winking and nudging each other, as if they were seeking to pass messages to each other. In discussion, it emerges that Maya’s parents have developed the idea that she can read their thoughts. They seek to test this, and wink and nudge each other as cues. However, while they are happy to discuss their ideas with the interviewers, when Maya asks them what they are doing, they deny that anything strange is going on.

In this case, the claims made by Laing and Esterson seem very plausible. Maya’s ideas appear delusional when she is considered outside of her family environment. However, when she is seen with her family, it makes sense that Maya would have the odd ideas that she does. In the family setting, the problems faced by Maya and her family do not seem to be caused by some dysfunction within her.

### Social models of disability

Social models of disability will likely be familiar to most readers and will be described only briefly here. The basic idea is that disability is caused by deficiencies in the social or material environment rather than by the biological deficiencies of those with different bodies [15, 16]. The classic example is the disability of a wheelchair user. There is a problem; the wheelchair user faces difficulties getting around. On the usual (medical) approach, the problem is located in the disabled woman’s body—the problem is her lack of legs. On the social model, on the other hand, the problem is instead located within the material and social environment—the problem is the lack of ramps. As another example, the communication problems faced by deaf people can be considered analogously,

From a medical and rehabilitative point of view (which is also the point of view of most hearing people), a deaf child is disabled by her inability to hear, and so the child becomes the focus of efforts to ‘normalize’ her as far as possible within the hearing community. But from another, equally valid point of view, the same child is handicapped by hearing people’s (often including her parents’) ignorance of Sign. [17, p. 29]

In the social model of disability, the basic idea is that disability should not be located within the individual person, because within a suitable environment the person would not have problems.

### Accounts of mental disorder derived from extended cognition approaches

In their classic 1998 paper, ‘The Extended Mind’, Andrew Clark and David Chalmers argue that the mind is not contained within the skull but can extend into the world [18]. They note the extent to which we think via ‘epistemic actions’, i.e., acting in the world. One of their examples concerns someone playing the computer game Tetris (where falling blocks have to be rotated and slotted into gaps in a wall). They note that some players rotate the blocks mentally, others use the computer controls and rotate on screen. In Clark and Chalmers’s view, it makes little difference where the reasoning is done; the player who rotates on screen is thinking in the world. Following their work, the idea that thinking may be extended has become a popular, if not universally accepted, view.

If the mind can be extended into the world, then it becomes plausible to think that certain kinds of mental disorder may be sited in the world too. Little work has been done so far on extended mental disorder,^3^ but it is easy enough to sketch an extended account for at least some cases. Indeed, in their original paper, Clark and Chalmers discuss a person with brain-related memory problems who uses a notebook to record matters to be remembered. Insofar as the notebook can fulfill the function of the damaged brain-related memory, they say that the notebook can be considered to be part of that human’s mind. In so far as beliefs and memories can be extended, this raises the possibility that when problems arise, these may be grounded in events external to the brain. Rather than seeking atrophied brain regions, we might need to look for destroyed diaries.

Clark and Chalmers’s notebook user is a thought experiment, but plausibly, there are some actual people whose memories are usually in the external environment. Deterioration often occurs when patients with dementia are moved from their homes [21, p. 139]. Dementia patients often arrange their own homes to support their thinking—needed items may be available to view, phone numbers and photos may be posted on walls, and so on. Dementia patients do, of course, have problems in their brains, but quite plausibly, the sudden deterioration seen in the moved patient is not caused by a worsening in their neurological condition. Rather, the deterioration can be better explained in terms of their being uprooted from the external system that contained their memories and supported their thinking.

There might also be other types of cases where an extended approach to mental disorder makes sense. Many people learn to control anger by walking away from problems. This normally works. But, in some environments, there is nowhere to walk. Anger-management issues are seen as particular problems in prisoners.

One of the big problems is that in prison, where there are many men who know that they must walk away from trouble when they see it brewing, there is simply no place to go. In a bar in a community, a man who knows he has trouble avoiding fights when he feels disrespected has to learn to get up and leave when someone starts insulting him or threatening him. [22, p. 719]

Often, anger problems in prison are thought of as being caused by faults within the angry prisoners—there is thought to be something wrong with their brains or psychology. But the difficulties could instead be seen as rooted in the environment. The problem here is that there is nowhere to walk away to.

## Shifting focus: how to locate a problem in a complex system

My aim is to assess the three ‘externalising’ accounts briefly outlined above. First, though, I want to switch to thinking in more abstract terms. How, in general, do we locate a problem when a complex system fails to function? Suppose there is some multiple-component system that is not working. Take, for example, a kettle that has been plugged in but fails to boil water. The problem might lie in the kettle, or it might be a problem with the socket. How does one locate the problem?

In such cases, we tend to swap components around. We try plugging the kettle into another socket. If we then get hot water, we conclude that the kettle is okay. Conversely, if we can get a different kettle to work in the original socket then we conclude that the socket is working. When the kettle works in a new socket, it is sometimes tempting to conclude that the original socket must have been faulty, but that would be a mistake. Such a conclusion would be unjustified because there can be ‘misfit’ problems, e.g., the kettle is fitted with a two-prong plug but the socket has three holes. In such cases, there is nothing wrong with the kettle; it will work in a different socket. But there is also nothing wrong with the socket; it will work with a different kettle.

## Evaluating the three accounts

I will consider Laing and Esterson’s argument first. Their reasoning is somewhat different from that of the proponents of the social model of disability, and the extended model of mental disorder. Laing and Esterson’s arguments, I shall argue, must ultimately be rejected because an essential step in their argument is missing.

In Laing and Esterson’s book, the implicit argument behind the claim that schizophrenia should not be thought of as a problem within the diagnosed women seems to be roughly as follows: Laing and Esterson first speak to the woman in isolation from her family. She appears deluded and has very odd beliefs. They then speak to the woman in her family context. Their perceptions shift. While there still seem to be problems (these are not happy and well-functioning families), the problem no longer seems to be with the ‘patient’. In the family context, the woman no longer appears irrational; rather, the problem appears to be with her wider family. The tacit reasoning going on here, I suggest, is that Laing and Esterson think that they themselves would do no better if forced to live with the woman’s family. They also assume themselves to be ‘normal’, ‘rational’ people. Laing and Esterson thus reason that if the patient were replaced by a test ‘normal’ subject there would still be a problem. Thus, they conclude the problem is not within the woman.

I suggest that this argument is invalid. Going back to my kettle-socket example, recall that there was a kettle that was plugged into a socket, but there was a problem and the water would not boil. Suppose that original kettle is replaced with a test kettle that is known to work. There is still a problem, however, and there is no hot water. In this case, can we conclude that the original kettle was functional and that the problem must have been in the socket? I suggest we should not. The problem is that both the socket and the original kettle might be faulty. Showing that there is still a problem even with the kettle replaced is not enough to show that the original kettle was functioning. To show that the original kettle was okay, we would also need to find a socket in which it works.

Analogously, I think there is a crucial step missing in Laing and Esterson’s argument. The possibility that they fail to rule out is that there is a problem both with the patient’s family and also within the patient. Before concluding that there is no problem with the patient, we also need to consider whether she would do okay in some other environment (as we should only conclude that the kettle is functioning if we can find some socket in which it works).

Here, things become unclear. Laing and Esterson say a lot about how the women they study function in their family context, but they say very little about how they do in other environments. The information they provide suggests that the diagnosed women do not actually do very well when placed in a different setting. Maya, the woman discussed earlier, for example, had been hospitalised for nine of the preceding ten years.^4^ For this reason, I suggest that Laing and Esterson’s argument fails. They fail to show that there is no problem within the women diagnosed with schizophrenia because they do not show that these women would function adequately in another environment.

Although I suggest that Laing and Esterson’s argument fails, there are some other mental disorder diagnoses where an analogous argument might plausibly go through. Conduct disorder is a psychiatric diagnosis that can be given to children who engage in a range of antisocial behaviour [12, p. 469], such as fighting, truancy, theft, rape, and so on. Now, consider a child who meets diagnostic criteria for conduct disorder but who lives in a poverty-stricken and gang-infested environment. In certain circumstances, it might be that any ‘normal test subject’ who is placed in that setting would act similarly to the diagnosed child. If I were hungry and threatened then I might also engage in theft, pre-emptive fighting, truancy, and so on. In some cases, it might also be the case that if he were placed in a different environment, the diagnosed child would stop behaving ‘symptomatically’. In a nice foster home, where children are given food and aggression is unnecessary, he would stop fighting and shop-lifting. In this sort of case, I suggest, it is reasonable to think there is nothing actually wrong with the child. But, here, in this case, where the argument goes through, it is only justifiable to conclude that there is no problem within the child because I am supposing the putative patient would function satisfactorily in a different environment.

There may also be situations in which a person, initially ‘normal’, comes to be damaged by a hostile environment, and is then unable to function ‘normally’ even once removed from that environment. Functioning kettles are seldom harmed by being plugged into the wrong type of socket, and will boil water as soon as they are plugged into the right socket. But a child might be brutalised by early experiences, and then behave in a disordered way even when placed in a nurturing environment. This would be a case in which an internal dysfunction is produced by a hostile environment. In environmentally caused disorders, there is (now) a problem within the patient. Experientially induced phobias and post traumatic stress disorder are clear examples of such environmentally caused disorders. If one thought that schizophrenia could be caused by dysfunctional family situations (in the sense that problematic family dynamics could cause internal cognitive dysfunction in an otherwise potentially normally developing child), then schizophrenia would also count as an environmentally caused disorder. Note that Laing and Esterson’s account of schizophrenia is distinct from such a view. Their claim is not that schizophrenia is caused by families but located in individual patients (they explicitly state that their claim is not ‘that the family is a pathogenic variable in the genesis of schizophrenia’ [1, p. 12]), but that schizophrenia is not a condition to be located within patients at all.

In contrast to externally caused disorders, the focus of this article is on cases in which a putative disorder should not be located within the ‘patient’ because there is no internal problem. The earlier case of ‘conduct disorder’, in which a child behaves normally when placed in a satisfactory environment, is my first example of such a case. I return now to the arguments made by the proponents of the social model of disability, and of extended models of mental disorder. Do these arguments work? In these accounts, the claim is that when the ‘patient’ is placed in a different environment, there is no problem, and that thus there is nothing wrong within the ‘patient’. Advocates of the social model say that since there would be no mobility problem in an environment with more ramps, disability should not be located within the individual wheelchair user. The angry man who cannot control his temper in prison may be fine in his home situation, where he would be able to walk away from provoking situations. In cases where putative patients function satisfactorily in a better environment, it seems fair to conclude that any problems should not be located ‘within’ them.

Can we conclude that in these cases, the problems lie in the inadequate environment (as the social model claims regarding disability, for example)? Is there something wrong with an environment that contains stairs rather than ramps? I suggest we should not claim this. This is because the environment in which the wheelchair user struggles works fine for many other people; many people can get around an environment with stairs. Rather than locating the problem within either the wheelchair-using individual or the stair-laden environment, I suggest that we can better conceptualise the problem as being caused by a mismatch. There is nothing wrong with a two-pronged plug or a three-pronged socket, but they do not work well together. Similarly, there is nothing intrinsically wrong with either the stairs or the wheel-chair user; it is just that they do not fit well together. The idea that disability is often best regarded as a misfit between an individual’s body or psychology and the environment has previously been argued for (on different grounds) by Rosemarie Garland-Thomson [24]. As she puts it, ‘The problem with a misfit, then, inheres not in either of the two things but rather in their juxtaposition, the awkward attempt to fit them together’ [24, p. 593]. She goes on: ‘The relational and contingent quality of misfitting and fitting, then, places vulnerability in the fit, not in the body’ [24, p. 600].

So far, I have spoken as if we should conclude that a disorder is not ‘in’ a putative patient if there is some environment or other in which there is no problem. I suggest, however, that this formulation is too permissive. Let me return (for the final time) to the kettle-socket example. It seems reasonable to conclude that there is nothing wrong with an electric kettle if there is some type of socket or other where it can be plugged in and will boil water. On the other hand, for us to conclude that an electric kettle has nothing wrong with it because it will boil water if placed in a fire seems wrong. Plugging the kettle into a different socket is a reasonable environmental shift, but getting the electric kettle to boil water by placing it in a fire is cheating!

Analogously, I suggest that there must be some limit to the range of ‘test’ environments that are appropriate when we argue that there is no problem within a putative patient because there is some environment in which they can function acceptably. Here, the phrase ‘reasonable adjustment’ is telling. I think that there must be some limit to the range of environments that count as fair adjustments. Arguing that someone has nothing wrong with them because they can gain nutrition in an environment that includes a gastric feeding tube or because they are mobile in an environment that includes hoists and lifts, will not do. However, as debates regarding what counts as a ‘reasonable adjustment’ make very clear, determining which kinds of alternative environments should be considered reasonable tests will depend on complex practical, moral, and political decisions. Exactly how these limits should be determined would be a task for another article. Here, I shall merely note that determining the limits is at least in part an ethical and political issue, and will necessarily be contested.

To conclude, I have argued that problems should not be located within an individual putative patient in cases where there is some acceptable test environment in which there is no problem. I have discussed a number of cases in which such an argument plausibly works. There may not be a problem ‘within’ a child diagnosed with conduct disorder, if they would behave better in a less violent environment. Similarly, an ‘angry man’ who can control his anger in an environment where he is able to walk away from problems is not disordered. Those disabilities that can be dealt with via ‘reasonable adjustments’ should similarly not be situated within individual bodies. However, Laing and Esterson’s argument that schizophrenia is not within diagnosed patients does not work. The problem with their argument is that they fail to show that the diagnosed women they consider function adequately when removed from their families.

## Footnotes

1 See, e.g., [3–5].

2 Laing also makes this claim in [14].

3 But see [19, 20].

4 In other work, Laing writes that the social context of mental hospitals is often ‘miserable’ and ‘mystifying’ [14, p. 47]. He would likely not consider the hospital to be a setting in which a ‘functioning’ person could be expected to behave ‘normally’. Still, the onus is on Laing to make it plausible that there is some other environment in which the women he interviews can function adequately, and in *Sanity, Madness and the Family*, he fails to do this. In a later essay, Laing writes of the success of ‘households’ that have been ‘operative in London run by those who stay in them’ [14, p. 46]. Here, he has in mind Kingsley Hall, a therapeutic community that ran from 1965 to 1970. Laing writes that ‘This experiment (currently on-going) has shown conclusively that many who behave in typically schizophrenic ways in some places behave differently in these households’ [14, p. 46]. Unfortunately, Laing wrote little about Kingsley Hall, and it is difficult to gauge how successful it was. Its most famous resident, Mary Barnes, eventually recovered from her psychotic episode and wrote about her experiences, but her recovery took a long time, and clearly her problems were not merely externally located [23].
